# The Effects of Stocking Density and Distances on Electroencephalographic Changes and Cortisol as Welfare Indicators in Brahman Crossbred Cattle

**DOI:** 10.3390/ani11102895

**Published:** 2021-10-05

**Authors:** Ahmed A. Abubakar, Idrus Zulkifli, Yong M. Goh, Ubedullah Kaka, Azad B. Sabow, Elmutaz A. Awad, Jurhamid C. Imlan, Azalea H. Othman, Razlina Raghazli, Helen Mitin, Awis Q. Sazili

**Affiliations:** 1 Institute of Tropical Agriculture and Food Security, Universiti Putra Malaysia, UPM, Serdang 43400, Selangor, Malaysia; ahmadsadeeq7@gmail.com (A.A.A.); zulidrus@upm.edu.my (I.Z.); elmutazatta@upm.edu.my (E.A.A.); jurhamidimlan@yahoo.com.ph (J.C.I.); helenmitin21@gmail.com (H.M.); awis@upm.edu.my (A.Q.S.); 2 Department of Animal Science, Faculty of Agriculture, Universiti Putra Malaysia, UPM, Serdang 43400, Selangor, Malaysia; 3 Department of Preclinical Sciences, Faculty of Veterinary Medicine, Universiti Putra Malaysia, UPM, Serdang 43400, Selangor, Malaysia; razlina81@gmail.com; 4 Department of Companion Animal Medicine and Surgery, Faculty of Veterinary Medicine, Universiti Putra Malaysia, UPM, Serdang 43400, Selangor, Malaysia; dr_ubedkaka@upm.edu.my; 5 Department of Animal Resource, College of Agriculture, Salahaddin University-Erbil, Erbil 44002, Kurdistan Region, Iraq; azad1979sabow@yahoo.com; 6 Department of Animal Science, College of Agriculture, University of Southern Mindanao, Kabacan 9407, North Cotabato, Philippines; 7 Department of Veterinary Pathology and Microbiology, Faculty of Veterinary Medicine, Universiti Putra Malaysia, UPM, Serdang 43400, Selangor, Malaysia; azalea@upm.edu.my; 8 Department of Veterinary Services, Wisma Tani, Blok Podium, Federal Government Administrative Centre, Putrajaya 62630, Malaysia; 9 Halal Products Research Institute, Universiti Putra Malaysia, UPM, Serdang 43400, Selangor, Malaysia

**Keywords:** Brahman crossbred cattle, cortisol, electroencephalographic, hot humid, road transport, Malaysia

## Abstract

**Simple Summary:**

The current study investigated the effects of stocking density and distances on electroencephalographic changes and cortisol as welfare indicators in Brahman crossbred cattle. The animals were transported by road for either 450 km or 850 km. After transportation by road, the animals were kept in lairage for 8 h. Both distances and stocking density affected various EEG parameters and cortisol results.

**Abstract:**

The objective of the current study was to investigate the effects of stocking density and distances on electroencephalographic changes and cortisol as welfare indicators in Brahman crossbred cattle. Sixty Brahman crossbred heifers were subjected to road transport from a cattle feedlot farm located in Universiti Putra Malaysia (UPM), Serdang to a commercial ruminant abattoir in Shah Alam, Selangor. Animals were assigned to long (850 km) and short (450 km) distances and high (600 sqm), medium (400 sqm), and low (200 sqm) stocking densities. Results revealed that the intensity of cortisol responses and EEG parameters (such as alpha <0.001, beta < 0.001, delta < 0.001, theta < 0.001, MF < 0.001 and Ptot < 0.001) increased significantly. Long-distance transport also resulted in significantly more intense (<0.001) responses to nociception during slaughter than animals that had been transported over a shorter distance, as indicated by EEG and cortisol.

## 1. Introduction

The transportation of animals for slaughter is a stressful event that has lasting effects on animals’ well-being and meat quality [1]. Transport is an essential and critical step in the meat production system. Journey distance and duration are significant factors that may affect animal welfare during transport from the farm to the slaughterhouse [1,2,3]. Food animals are transported all over the world due to the strength and specialisation of livestock production in specific areas, as well as the need for them to be sold and slaughtered in locations where they are not grown [4,5]. Transport induces stress on animals, and these stress factors can be categorized into two categories; the “short-acting” factors that tend to have emotional effects on animals and the “long-acting” factors that have physical consequences and may accumulate over time [6].

The negative impacts of transport on meat quality and the associated economic losses are the primary reasons [2,7] behind the driving necessity to reduce stress-inducing factors associated with transportation [8]. Findings have shown that transport duration and distance (as well as stocking density) affect the meat quality and well-being of cattle [9,10], lambs [11,12,13], pigs [14,15] and rabbits [16]. However, numerous studies have been conducted on the on-road transportation and welfare of farm animals in temperate climates. On the other hand, here in Malaysia, previous studies conducted on the road transport of farm animals were focused on goats [17], poultry [18,19] and rabbits [20].

When animals are exposed to stressful conditions, they respond by activating their sympathetic nervous system. Animals react to stressful situations or stimuli by releasing stress hormones into the bloodstream [21]. Most often, hormone based techniques have a time lag following a stress-induced change; a technique assessing instantaneous response will be vital to animal welfare. Electroencephalography is an established tool used in recording the instantaneous physiological response to nociception in animals. An electroencephalogram (EEG) involves placing electrodes in different positions on the scalp to monitor electrical activity [21]. Electroencephalogram spectrum changes have been used as an indicator to measure animals’ responses to nociception in sheep [22], goats [23], cattle [24,25], dogs [26,27] and horses [28,29]. All of these studies used EEG to measure changes in the EEG spectrum in response to nociceptive stimuli. So far, literature regarding EEG changes in response to non-noxious stressors is scarce. 

According to Moberg’s stress model (2000) [30], when an animal perceives a threat, the central nervous system (CNS) develops a combination of four general biological defence responses, which consists of some behavioural, autonomic nervous system, neuroendocrine and/or immune responses. The electroencephalogram is a real-time graphical representation of tiny, spontaneously generated electrical currents of neurons (in the microvolt range) from the cerebral cortex through electrodes located at different locations on the scalp in humans or the head in other species [21]. We hypothesize that this non-noxious stressor may induce changes in the EEG spectrum, similar to some painful stimuli.

Data on the influence of distances and stocking density during road transportation in cattle, especially under hot, humid tropical conditions in Malaysia, are scarce. The exception to this are the most recent studies, which studied the effect of sea and road transport of farm animals [31]. These call for a study to assess the impact of road transport on farm animals, the use of which is on the rise due to high demand and the welfare problems associated with handling and thermal extremes. There’s a pressing need to establish a common standard and guideline on transportation of cattle in light of the enforcement of the Animal Welfare Act (2015) [32]. These require thorough scientific data to formulate a guideline for livestock handling, transportation and management. Such detailed scientific data can only be obtained through the conducting of a rigorous field animal trial. 

Thus, the present study was undertaken to investigate the effects of stocking density and distances on electroencephalographic changes and cortisol as welfare indicators in Brahman crossbred cattle. These changes are crucial to understanding how cattle respond to transportation stress during short and long-distance transport. Furthermore, it is vital to know whether the intensity of transport stimuli would affect cattle’s response to noxious stimuli at slaughter and other welfare concerns that determine meat quality in beef-producing animals.

## 2. Materials and Methods 

### 2.1. Ethical Approval 

The Institutional Animal Care and Use Committee (IACUC) of the Universiti Putra Malaysia approved the protocols for the current trial (UPM/IACUC/R028/2016). 

### 2.2. Housing and Management 

The animals were kept in naturally ventilated pens (15 animals per pen) fitted with a concrete floor and PVC roofing. The space allowance was 3.5 m^2^/animal. Animals had access to commercial beef cattle feed, grass pellets, rice straw, drinking water ad libitum and lighting for 24 h. The average in-house temperatures recorded were 33.0 ± 1.36 °C during the day and 23.1 ± 1.40 °C at night, and the relative humidity recorded was 82.6 ± 1.40% (The Malaysian Meteorological Department) [33].

### 2.3. Animals, Transport, and Treatment 

A total of sixty (60) heifers (Brahman crosses) of about 24 months of age with a live weight of about 290.0 ± 36.0 kg were allotted to two different distances (*n* = 30) 450 km (short-distance) and 850 km (long-distance) and were further subdivided into three different stocking densities (*n* = 10) (i.e., 600 kg/sqm (high-SD), 400 kg/sqm (medium-SD) and 200 kg/sqm (low-SD)). Before starting the experiment, randomisation was ensured using a random number assignment, and all animals used were homogenous. All animals were hauled for either 9 h (short-distance) and or 17 h (long-distance) prior to unloading at a commercial abattoir for slaughter. The time for both departures and arrivals at the slaughter plant were recorded. The highway was used to transport all animals. The duration of transport for randomly selected animals was used in calculating the differences between arrivals and departure times. The average daily temperatures at the farm and other parts of the state the animals cross during transportation ranged between 32 and 35 °C during the day and 22 and 24 °C at night. Relative humidity was (84.1%); all the data were obtained from the Malaysian Meteorological Department [33]. 

### 2.4. Blood Sampling 

As a precaution, cleaning was done aseptically and samples were collected via the jugular venepuncture using an 18-gauge needle and a 10-millilitre blood collection tube for serum and plasma (with heparin as an anticoagulant) (BD Franklin Lakes, NJ, USA). The samples were collected before the animals were loaded T1 (at the farm as baseline values while the animals were in their peer-groups), T2 (within 30 min after arriving at the abattoir) and at slaughter T3 (post-neck cut from blood flow), which were kept on ice temporarily. Centrifugation was done for serum samples at a speed of 1600× *g* at a temperature of 8 °C for 15 min which were later stored at −80 °C until subsequent analysis.

### 2.5. Slaughtering Procedure 

The slaughtering procedure was carried out at the large ruminant’s section of the Abattoir, Department of Veterinary Services in Shah Alam, Selangor Darul Ehsan. On arrival, animals were unloaded carefully within 30 min and rested in the lairage area for 12 h, where water was provided freely before slaughter. After the lairage, animals were carefully transferred and conveyed into the restraint box (mark4 box). All animals were humanely slaughtered without stunning following the dictates of the Malaysian standards MS1500: 2009 (Department of Standards Malaysia, 2009) [34]. The process involves severing the two carotids, jugular veins, trachea and oesophagus.

### 2.6. Electroencephalogram Recording 

The electroencephalographic activity was recorded using a Power Lab Bio-potential Recording System (AD Instruments, Bella Vista, NSW, Australia) at four-time points. Namely, before loading the cattle for transport (T1), within 30 min after unloading at the abattoir (T2), in a modified Mark IV box while being prepared for slaughter (T3), and immediately after neck-cut during slaughter (T4). Ten animals for each distance and stocking density combination were randomly selected for the EEG recording on each occasion. The distances and stocking density combinations were comprised of long-distance high-stocking density (SD), long-distance medium-SD, long-distance low-SD, short-distance high-SD, short-distance medium-SD and short-distance low-SD groups. The electroencephalogram (EEG) recording was performed using disposable surface electrodes (Covidien LLC, Hampshire Street, Mansfield, QC, Canada). Before the recording area was shaved and cleaned with 70% alcohol, the inverting negative electrode (−) was attached 6–8 cm distally from the poll, equidistant to both of the anterior orbital prominences of the left and right eyes. The second non-inverting positive electrode (+) was attached on the mastoid process. The electroencephalographic signal was recorded at a sampling rate of 1 kHz. Raw EEG was re-sampled with a low pass filter of 200 Hz into delta frequency (0.1 to 4 Hz), theta frequency (4.1 to 8 Hz), alpha frequency (8.1 to 12 Hz) and beta frequency (12.1 to 20 Hz). Analysis of the EEG data was performed offline after completing experiments using the Chart Spectral Analysis Function (Chart 5.0TM software Instruments, Bella Vista, NSW, Australia). Before analysis, potential interferences from the concurrent electrocardiograph signals were digitally removed from the raw EEG recordings using the Chart 5.0TM software. The signals were then processed for 60 epochs per minute in non-overlapping 1-s epochs. The root mean square of the alpha, beta, delta and theta waves at T1, T2, T3 and T4 were calculated once subjected to the fast Fourier transformation procedure. Median frequency (F50, the frequency below 50% of the total EEG Power) and total power (Ptot, which is the total area under the power spectrum curve) were also determined.

### 2.7. Determination of Cortisol

The quantitative analysis of bovine cortisol concentrations using serum/plasma was determined using an Enzyme-Linked Immuno-Sorbent Assay (ELISA) kit #MBS701325 from My-BioSource San Diego, CA, USA. The kit operates based on inhibiting the competition between the pre-coated cortisol and the cortisol in samples. The analysis was achieved by complying with all the manufacturer’s protocols as contained in the kit’s manual. The intra- and inter-assay variabilities were less than 8% and 10%, respectively, and the detection range was 0.049–200 ng/mL. 

### 2.8. Statistical Analysis

All datasets were tested for their conformity to the assumption of normality using Shapiro–Wilk’s procedure before the analysis. The experiment followed a 2 × 3 (distance × stocking density) factorial model, taking into account the sample size across treatment groups, and analysed for changes across four-time points for EEG and three-time points for cortisol, using the repeated measure analysis variance (RM-ANOVA) approach. The GLM procedure was used in analysing all datasets of the statistical analysis system (SAS) package Version 9.4 software [35], in which stocking density, distance and interactions were examined. Means that were significantly different were interpreted using the LSD multiple range test. All statistical analysis was performed at a 95% confidence level. Sample size calculation was performed using G*Power 3.1 software [36,37]. The calculation was performed using the standardised difference (or effect size) derived from significant parameters measured in the current experiment at the recommended test power (1-beta) of 80% as recommended by G*Power. The extent of fluctuation in sample size was also determined at a test power of 70% and then 90% to minimise Type 2 errors.

## 3. Results

Table 1, Table 2 and Table 3 show that all treatment groups had similar starting values for their EEG parameters across all waveforms and their power spectrums at T1. It is also evident that the introduction of stimuli at T2, T3 and T4 resulted in a significant increase in the RMS, Ptot, and median frequency (F50) for all experimental groups (except the median frequency (F50)) among animals transported over a 450 km distance (Table 3). The most notable changes across T1 to T4 were for beta waves (Table 1), Ptot and F50 (Table 3), where a significant interaction between distance × stocking density was observed. 

Results on the influence of distances and stocking density on Cortisol concentration are shown in Table 4, Table 5 and Table 6.

Table 5 below shows the interaction between handling as influenced by stocking density in cattle subjected to road transport. Our findings confirmed a similar trend observed by other researchers, with a mean pre-load value in animals subjected to different stocking densities of 51.29 ± 0.87 for 200 kg, 51.30 ± 1.06 for 400 kg and 51.85 ± 0.78 ng/mL for 600 kg, unloading for animals stocked at 108.19 ± 6.41 for 200 kg, 121.36 ± 5.31 for 400 kg and 134.62 ± 8.28 for animals stocked at 600 kg respectively. Animals stocked at 200 kg had values of 140.69 ± 1.44, animals stocked at 400 kg had values of 159.02 ± 2.51 and animals stocked at 600 kg had values of 172.90 ± 3.87 ng/mL at slaughter. Furthermore, as shown in Table 4, there was a significant interaction between handling and stocking density.

Table 6 below shows the interaction between handling as influenced by distance in cattle subjected to road transport. Our results showed a similar trend observed by other researchers, a mean value of 51.97 ± 1.30 ng/mL as the baseline value before loading T1 in animals subjected to a distance of 450 km, 108.16 ± 5.70 ng/mL during unloading and 151.22 ± 3.32 ng/mL during neck cut T3 were recorded. Similarly, long-distance animals had mean values of 51.13 ± 0.73 ng/mL T1 (before loading), 134.62 ± 4.96 ng/mL for T2 (unloading), and 163.85 ± 2.62 ng/mL for neck cut. As demonstrated in Table 4, there was an interaction between handling and distance.

## 4. Discussion

When discussing this study’s results, it should be noted that this is the first study on cattle using EEG and cortisol to assess animals’ welfare subjected to transport by road with different stocking densities and distances within the hot, humid tropical region of Malaysia. These findings are in accord with the previous work by Zulkifli et al. (2014) [24] on the presence of noxious stimuli among animals that had been subjected to non-penetrative stunning based on changes in F50 and Ptot, in conjunction with changes observed for delta and theta RMS. These changes had earlier been confirmed by Murell and Johnson [21] and Gibson et al. [25] to indicate noxious pain. In the present study, EEG evidence among animals transported over a long distance suggested that these animals probably experienced significantly more intense noxious stimuli at slaughter regardless of the stocking density. It is an important finding that is yet to be reported for large ruminants.

The current study results are associated with the animals’ ability to adapt to transportation stress (T2) and how they respond to noxious stimulus during slaughter (T4). Electroencephalography is an established technique used in recording the instantaneous physiologic response to stress and nociception (pain) in animals [19,20]. It is as essential as biochemical and hormonal based methods, often exhibiting lag time following a stress-induced change. Generally, alpha and beta waves indicate cognitive and stress changes in a fully alert and awake state. In contrast, changes to both delta and theta waves (the so-called slow waves) are associated with changes in the state of consciousness. These slow waves would also increase as the Ptot of an EEG spectrum increases; for example, during stressful episodes. However, should these slow waves dominate the EEG power spectrum, it is probably indicative of a loss of consciousness or even a rapid onset of brain pathologies. The Ptot of an EEG spectrum is also vital as it indicates the intensity of an animal’s response to a stimulus. These should be interpreted alongside other indicators (such as Median Frequency (F50)). Spiking of F50 accompanied by a reduction of the intensity of slow waves is typically associated with the presence of a noxious stimulus [25,38].

Based on Table 1, it is evident that stocking density is an essential determinant of stress response in animals. Stocking densities are exceeding 400 kg/sqm, which induced spiking EEG changes consistently among cattle transported over a long distance in the current trial. Transporting animals at higher stocking density over longer distances (850 km) is significantly more stressful, as shown by the beta wave of the long-distance high-SD animals (Table 1, T2). However, the T2 Ptot values from Table 3 showed the opposite trend. Animals transported over long distances had significantly lower (*p* < 0.05) Ptot at T2 than did animals undergoing short-distance transportation. The lower Ptot for animals transported over 850 km strongly suggests that these animals may have been slightly ahead in adapting to the transportation condition, while cattle subjected to a shorter distance (450 km) still adapt to transportation after-effects. On another note, the Ptot values at T3 and T4 for animals transported over shorter distances from Table 3 are consistent with a lower magnitude of response to noxious stimuli associated with slaughter. On the contrary, the Ptot values from animals transported over a long-distance where the F50 at T4 for two groups of animals transported over a long distance (high-SD and medium-SD) was more than 60% higher than their contemporaries that had been subjected to a shorter transportation distance. Taken together with the significantly lesser RMS values of the delta and theta waves (Table 2) at T4, this evidence suggested that cattle subjected to long-distance transport might have experienced considerably higher noxious stimuli at slaughter based on higher F50 values at T4 (Table 3). It is interesting to note that all T3 values for Table 1, Table 2 and Table 3 were not significantly different (*p* > 0.05) across distances and stocking densities. It is an important observation, supporting the fact that the 8 h lairage period implemented in the current experiment is adequate for the animals to adapt. Apart from the critical results highlighted, alpha wave (Table 1), delta wave (Table 2) and theta wave (Table 2) values are generally consistent with the findings that long-distance transportation at mid or high stocking densities induced a significant increase in EEG parameters.

A significant increase in EEG parameters after neck cut (T4) compared with pre-load (baseline T1) in all animals subjected to transportation by road with different distances and stocking densities were observed in the current study. It is worth mentioning that earlier works by researchers have reported the median frequency (MF or F50) and total power (Ptot) as useful indicators of pain [22,24,38,39]. In the current study, F50 increased significantly with the increase in distance and stocking density in both groups compared with baseline values. Similar results were reported in response to neck cut in goats [23], calves [25] and cattle [24]. These rendered pieces of evidence that transportation procedures could potentially alter the threshold to perceive noxious stimuli in animals at slaughter [40]. Kaka et al. (2016) [41] had demonstrated that preoperative manipulation in dogs could potentially change pain perception in dogs. Additionally, Gibson et al. (2009) [39] also report that operating procedures often result in a reaction to pain and hyperalgesia. Therefore, it is not surprising that the same phenomenon could have been possible in large ruminants, albeit with transportation distance as the determinant and an altered sensitivity to pain during slaughter as suggested in the current study. 

The electroencephalogram has been used as a vital tool in assessing unconsciousness, as it is the underlying electrical activity of the neurons population (which is supported by glial cells [21]). EEG indicates characteristic changes in unconscious animals. Analysis of these changes (EEG) gives an insight into the degree of nociception and stress animals experience at the point of neck cutting [40]. EEG detects the perception of noxious stimuli in the brain, which typically begins with arousal and desynchronization. Desynchronization is a typical EEG characteristic in humans [29,30]. Alpha waves are thought to signify calm consciousness without any focus or concentration in humans. Beta waves are the brain’s normal waking rhythm, which is correlated with active thought, active participation and concentration on the outside world in adults. When people are in a panic condition, their beta waves are stronger [21,42]. Delta waves are associated with deep sleep. Access to unconscious content, creative inspiration and deep meditation have all been linked to theta waves [43].

Plasma cortisol is an essential adrenal glucocorticoid secreted by cattle due to stress [9]. According to Broom (2003) [9], stress is an environmental effect on an individual that overstretches its control systems and reduces its fitness or appears likely to do so. Results suggest that transport stress induces an increase in the hypothalamus–hypophysis–thyroid axis activity, together with the peripheral tissue request, which is evident during short-distance transport and which continues to increase after long-distance transport. Our results showed that cortisol concentrations increased from a pre-loading mean value of 51.48 ng/mL through the procedures of loading and transporting animals under hot, humid tropical conditions. Cortisol levels remain elevated by about twofold, reaching as high as 121.39 ng/mL immediately after being unloaded from the truck. Multiple factors (including psychological or physical aspects such as handling (loading and unloading) and exposure to the environment with high ambient temperature), could be responsible for the elevated levels found. A high-stress level induces dehydration due to water deprivation and urination, fear/arousal and novelty [44,45,46]. Similarly, our results indicate a sharp contrast between distances. A significant interaction was observed, with short-distance having a lower cortisol level of 103.74 and 116.54 ng/mL after a long-distance. 

Additionally, cortisol concentration differs between stocking density, with animals subjected to higher-SD having a mean cortisol value of 119.79 ng/mL, medium-SD having a mean value of 110.56 ng/mL, and low-SD having a mean value of 100.06 ng/mL, a significant interaction was observed. At the point of neck cut, cortisol levels elevate further by three folds with a mean value of 157.54 ng/mL, which may be associated with fear and novelty, stress due to handling while inside the restraint box, and noise from hydraulics as observed by numerous findings. The results observed are in line with those of [47], who reported an elevated cortisol level after long haul periods. The current study observes that road transport stress induces an increase in the hypothalamus–hypophysis–thyroid axis activity, together with the peripheral tissue request, which is evident during short-distance transport and which continues to increase after long-distance transport. Our results showed that cortisol concentrations increased from a pre-load mean value of 51.48 ng/mL through the procedures of loading and transporting animals under hot, humid tropical conditions. 

Additionally, cortisol concentration differs between stocking densities, with animals subjected to higher-SD having a mean cortisol value of 119.79 ng/mL, medium-SD having a mean value of 110.56 ng/mL and low-SD having a mean value of 100.06 ng/mL, respectively. At the point of neck cut, cortisol levels elevate further (threefold) with a mean value of 157.54 ng/mL, which may be associated with fear and novelty, stress due to handling while inside the restraint box and noise from hydraulics, as observed by numerous findings. The results observed are in line with those of elevated cortisol after long haul periods reported by Knowles and Warriss [47] and post-road transportation in cattle by Zulkifli et al. [31].

Cortisol values remained elevated by about two folds with values as high as 121.39 ng/mL immediately after unloading them from the truck. The higher levels observed could be likely due to multiple factors ranging from psychological or physical aspect such as handling (loading and unloading) and exposure to the environment with high ambient temperatures. A high-stress level induces dehydration due to water deprivation and urination, fear/arousal and novelty [44,45].

Furthermore, our findings agree with those of Fazio et al. and Earley and O’Riordan [45,48], who reported an increase in cortisol plasma concentration. Additionally, both reported an increase in cortisol plasma concentration. Tarrant [49] also discovered that as stocking densities increased, plasma cortisol concentrations increased [50,51]. Cortisol levels rise as distance increases, according to our findings (which may likely be due to factors such as thermal extremes that trigger the release of stressors into the bloodstream). Fear due to handling, novelty and increased adrenal activity predisposes the animals to welfare problems [44,45,46]. Similarly, Buckham and Sporer et al. [52] transported bulls by road for 14 h and recorded a dramatic elevation in cortisol. Our findings agree with the work by Fazio et al. [45], who reported an increase in cortisol with increased journey duration. However, it disagrees with Earley et al. [44], who observe no difference with increased distances and or journey duration. 

## 5. Conclusions

Long-distance transport is more stressful compared to short-distance transport regardless of stocking densities. Long-distance transport also allowed for the possibility for animals to partially adapt to the transportation condition. However, based on experimental evidence, which indicated that long-distance transport also resulted in a significantly more intense response to nociception during slaughter than in animals transported over a shorter distance, it is right that animals are transported over a long distance at a stocking density exceeding 400 kg/sqm. In summary, the current research showed that stocking density is an essential determinant for stress response over long transport distances. Pieces of evidence suggested that the pain threshold might have been lowered to the extent that animals exhibited a more intense response at the point of slaughter. 

## Figures and Tables

**Table 1:** Influence of distances and stocking density on the encephalogram of Brahman crossbred cattle (alpha and beta waves) (Mean ± SE).

Parameters	Time Points	Long-Distance (850 km)			Short Distance (450 km)			Level of Significance		
		High-SD (600 kg/sqm)	Medium-SD (400 kg/sqm)	Low-SD (200 kg/sqm)	High-SD (600 kg/sqm)	Medium-SD (400 kg/sqm)	Low-SD (200 kg/sqm)	Distance	Stocking Density	Distance × Stocking Density
Alpha Wave (uV)	-									
-	T1	1.122 ± 0.158 y	1.040 ± 0.118 y	0.966 ± 0.133 y	1.182 ± 0.144 z	1.100 ± 0.118 y	0.803 ± 0.141 y	0.900	0.170	0.642
	T2	0.977 ± 0.122 cz	0.812 ± 0.186 cy	0.974 ± 0.161 cy	1.665 ± 0.122 ay	1.406 ± 0.144 bx	0.984 ± 0.108 cy	0.001	0.470	0.025
	T3	2.462 ± 0.351 x	1.683 ± 0.234 x	1.395 ± 0.286 x	2.151 ± 0.351 x	1.647 ± 0.405 x	1.523 ± 0.314 x	0.788	0.608	0.049
	T4	6.061 ± 0.568 aw	5.964 ± 0.464 aw	4.931 ± 0.696 bw	5.351 ± 0.623 aw	4.218 ± 0.691 cw	3.380 ± 0.670 dw	0.015	0.518	0.047
-	*p*-Value	<0.001	<0.001	<0.001	<0.001	0.024	0.019	-	-	-
Beta Wave (uV)	-									
-	T1	1.707 ± 0.260 z	1.786 ± 0.229 y	1.848 ± 0.308 y	1.453 ± 0.202 z	1.831 ± 0.281 y	1.412 ± 0.281 y	0.720	0.179	0.321
	T2	2.950 ± 0.292 ay	2.465 ± 0.350 bx	2.215 ± 0.271 cx	2.400 ± 0.241 by	2.560 ± 0.301 bx	2.230 ± 0.277 cx	0.040	0.037	0.015
	T3	3.186 ± 0.586 x	2.864 ± 0.391 x	2.775 ± 0.478 x	3.184 ± 0.586 x	2.825 ± 0.677 x	2.747 ± 0.524 x	0.204	0.780	0.209
	T4	8.012 ± 0.677 w	7.594 ± 0.505 aw	6.107 ± 0.757 cw	7.335 ± 0.618 bw	7.132 ± 0.500 w	4.869 ± 0.670 dw	0.028	0.036	0.008
-	*p*-Value	<0.001	<0.001	<0.001	<0.001	<0.001	0.004	-	-	-

Time points description: T1: before loading the cattle for transport, T2: within 30 min after unloading at the abattoir, T3: inside the Mark IV box being prepared for slaughter and T4: immediately after neck-cut during slaughter. a, b, c: Means in the same row with different letters are different at *p* < 0.05. w, x, y: Means in the same column with different letters are different at *p* < 0.05.

**Table 2:** Influence of distances and stocking density on the encephalogram of Brahman crossbred cattle (delta and theta. waves) (Mean ± SE).

Parameters	Time Points	Long-Distance (850 km)			Short Distance (450 km)			Level of Significance		
		High-SD (600 kg/sqm)	Medium-SD (400 kg/sqm)	Low-SD (200 kg/sqm)	High-SD (600 kg/sqm)	Medium-SD (400 kg/sqm)	Low-SD (200 kg/sqm)	Distance	Stocking Density	Distance × Stocking Density
Delta Wave (uV)	-									
-	T1	6.495 ± 0.818 x	5.599 ± 0.610 y	5.993 ± 0.691 y	6.876 ± 0.747 y	5.436 ± 0.611 y	5.542 ± 0.702 z	0.661	0.147	0.854
	T2	5.100 ± 0.675 abxy	5.719 ± 1.031 bcy	4.581 ± 0.893 ay	5.328 ± 0.595 by	6.880 ± 0.799 cy	7.352 ± 0.675 dy	0.031	0.177	0.061
	T3	9.998 ± 2.595 x	11.079 ± 2.119 x	12.441 ± 2.178 x	13.639 ± 3.178 x	11.808 ± 3.010 x	12.572 ± 2.843 x	0.734	0.377	0.610
	T4	60.346 ± 4.392 aw	57.637 ± 4.036 abw	55.160 ± 4.928 abw	53.650 ± 4.392 w	49.680 ± 6.612 cw	47.783 ± 5.210 cw	0.039	0.328	0.432
-	*p*-Value	<0.001	<0.001	<0.001	<0.001	<0.001	<0.001	-	-	-
Theta Wave (uV)	-									
-	T1	1.483 ± 0.156 y	1.491 ± 0.137 y	1.484 ± 0.184 y	1.432 ± 0.168 y	1.481 ± 0.137 y	1.686 ± 0.160 y	0.538	0.557	0.500
	T2	1.335 ± 0.202 cy	1.376 ± 0.309 cy	1.567 ± 0.298 by	1.441 ± 0.179 bcy	1.643 ± 0.240 by	2.343 ± 0.240 ax	0.039	0.050	0.144
	T3	2.187 ± 0.370 x	2.826 ± 0.400 x	2.150 ± 0.302 x	2.182 ± 0.342 x	2.106 ± 0.303 x	2.348 ± 0.431 x	0.778	0.418	0.332
	T4	10.573 ± 0.924 w	9.669 ± 0.755 bw	10.248 ± 1.132 w	7.754 ± 0.813 cw	6.531 ±0.669 cw	6.996 ± 0.732 cw	0.001	0.029	0.213
	*p*-Value	<0.001	<0.001	<0.001	<0.001	<0.001	<0.001	-	-	-

Time points description: T1: before loading the cattle for transport, T2: within 30 min after unloading at the abattoir, T3: Inside the Mark IV box being prepared for slaughter and T4: immediately after neck-cut during slaughter. a, b, c: Means in the same row with different letters are different at *p* < 0.05. w, x, y: Means in the same column with different letters are different at *p* < 0.05.

**Table 3:** Influence of distances and stocking density on the total power (Ptot) and median frequencies (F50) on the encephalogram of Brahman crossbred cattle (Mean ± SE).

Parameters	Time Points	Long-Distance (850 km)			Short Distance (450 km)			Level of Significance		
		High-SD (600 kg/sqm)	Medium-SD (400 kg/sqm)	Low-SD (200 kg/sqm )	High-SD (600 kg/sqm)	Medium-SD (400 kg/sqm)	Low-SD (200 kg/sqm)	Distance	Stocking Density	Distance × Stocking Density
Total Power (Ptot, V^2^/Hz)	-									
-	T1	11.417 ± 1.532 y	11.230 ± 1.351 y	12.996 ± 1.522 y	13.139 ± 1.301 y	12.958 ± 1.655 z	14.122 ± 1.514 y	0.202	0.286	0.078
	T2	12.976 ± 1.666 cy	11.766 ± 1.227 cy	12.976 ± 1.539 cy	18.098 ± 1.666 ax	15.618 ± 1.470 by	15.085 ± 1.272 by	0.035	0.045	0.101
	T3	27.547 ± 4.980 x	25.629 ± 4.313 x	24.225 ± 3.838 x	20.148 ± 4.123 x	22.941 ± 3.521 x	22.333 ± 3.991 x	0.068	0.293	0.520
	T4	75.113 ± 3.390 aw	73.812 ± 4.126 w	70.917 ± 2.768 bw	63.228 ± 3.713 w	61.044 ± 4.161 cw	63.228 ± 3.705 cw	0.001	0.324	0.029
-	*p*-Value	<0.001	<0.001	<0.001	<0.001	<0.001	<0.001	-	-	-
Median Frequency (F50, Hz)	-									
-	T1	8.510 ± 1.669 y	9.919 ± 1.227 y	8.690 ± 1.833 x	10.135 ± 1.315	10.905 ± 1.460	9.124 ± 1.500	0.377	0.409	0.921
	T2	10.97 ± 1.992 ay	11.675 ± 1.208 ay	11.255 ± 1.493 aw	10.002 ± 1.332	9.727 ± 1.782	10.112 ± 1.088	0.680	0.451	0.275
	T3	13.359 ± 1.241 ax	13.312 ±2.055 ax	12.953 ± 1.054 abw	11.710 ± 2.101 ab	10.974 ± 1.802 b	9.912 ± 1.961 b	0.046	0.061	0.126
	T4	17.615 ± 1.353 w	16.008 ± 1.189 w	13.326 ± 1.460 cw	11.675 ± 1.444 cd	10.359 ± 2.011 d	9.981 ± 2.070 d	0.002	0.036	0.048
-	*p*-Value	<0.001	0.003	0.026	0.254	0.388	0.721			

Time points description: T1: before loading the cattle for transport, T2: within 30 min after unloading at the abattoir, T3: Inside the Mark IV box being prepared for slaughter and T4: immediately after neck-cut during slaughter. a, b, c: Means in the same row with different letters are different at *p* < 0.05. w, x, y: Means in the same column with different letters are different at *p* < 0.05.

**Table 4:** Cortisol, as influenced by handling, stocking density and distances in Brahman crossbred cattle.

Parameters	Cortisol
Main Effects	-
Handling	-
Before loading (T1)	51.48 ± 0.52 ^c^
Unload (T2)	121.39 ± 2.23 ^b^
Slaughter (T3)	157.54 ± 4.07 ^a^
Density	-
200 kg	100.06 ± 4.90 ^c^
400 kg	110.56 ± 5.56 ^b^
600 kg	119.79 ± 6.71 ^a^
Distance	-
450 km	103.74 ± 4.50 ^b^
850 km	116.54 ± 4.98 ^a^
*p*-Value	
Handling	<0.0001
Density	<0.0001
Distance	<0.0001
Handling × Density	0.0012
Handling × Distance	0.0002
Density × Distance	0.2820

^a–c^ Means within sub-group with no common lowercase letters are different at *p* ≤ 0.05. T1: before loading the cattle for transport, T2: within 30 min after unloading at the abattoir, T3: immediately after neck-cut during slaughter.

**Table 5:** Cortisol, as influenced by the interaction between handling and stocking density in Brahman crossbred cattle.

Parameter	Cortisol		
	Handling		
Density	Before Loading (T1)	Unload (T2)	Slaughter (T3)
200 kg	51.29 ± 0.87 cx	108.19 ± 6.41 by	140.69 ± 1.44 az
400 kg	51.30 ± 1.06 cx	121.36 ± 5.31 bxy	159.02 ± 2.51 ay
600 kg	51.85 ± 0.78 cx	134.62 ± 8.28 bx	172.90 ± 3.87 ax

a–c: Means within rows sub-group with no common lowercase letters are different at *p* ≤ 0.05. x–z: Means within columns sub-group with no common lowercase letters are different at *p* ≤ 0.05. T1: before loading the cattle for transport, T2: within 30 min after unloading at the abattoir, T3: immediately after neck-cut during slaughter.

**Table 6:** Cortisol, as influenced by the interaction between handling and distances in Brahman crossbred cattle.

Parameter	Cortisol		
	Handling		
Distance	Before Loading (T1)	Unload(T2)	Slaughter (T3)
450 km	51.97 ± 1.30 cx	108.16 ± 5.70 by	151.22 ± 3.32 by
850 km	51.13 ± 0.73 cx	134.62 ± 4.96 ax	163.85 ± 2.62 ax

a–c: Means within rows sub-group with no common lowercase letters are different at *p* ≤ 0.05. x–y: Means within columns sub-group with no common lowercase letters are different at *p* ≤ 0.05. T1: before loading the cattle for transport, T2: within 30 min after unloading at the abattoir, T3: immediately after neck-cut during slaughter.

## Data Availability

Not applicable.
